# Osteolytic myxopapillary ependymoma with marked hyaline degeneration in a 72-year-old male: A case report

**DOI:** 10.3892/ol.2013.1397

**Published:** 2013-06-14

**Authors:** HAI WANG, ZHIYUAN ZHANG, MOHAMMAD SHAHIDUL MAKKI, QUNLI SHI

**Affiliations:** 1 Department of Pathology, Jinling Hospital, Nanjing University School of Medicine;; 2 Department of Neurosurgery, Nanjing General Hospital of Nanjing Military Command, Nanjing, Jiangsu 210002, P.R. China;; 3 Department of Molecular and Cellular Oncology, The University of Texas MD Anderson Cancer Center, Houston, TX 77030, USA

**Keywords:** myxopapillary ependymoma, hyaline degeneration, immunohistochemistry

## Abstract

Myxopapillary ependymomas (MPEs) are uncommon and account for ∼15% of all ependymomas. The current study presents a case of rare spinal MPE with abnormal hyaline degeneration. The patient was a 72-year-old male with a 10-month history of lower back pain. Magnetic resonance imaging revealed a mass involving the L4 and L5 vertebrae with local bone destruction. The tumor was completely resected. Histologically, the majority of the tumor exhibited low cellularity. A marked change in hyaline was observed in the blood vessels and stroma. In specific areas, the tumor showed reticular or tubular patterning embedded in hyaline materials. The tumor cells were cuboidal to columnar in shape with strong immunostaining for glial fibrillary acidic protein and S-100. A fluorescence *in situ* hybridization analysis for amplification of the epidermal growth factor receptor gene was negative. The results of pathological and immunohistochemical studies were consistent with the ependymal nature of neoplastic cells.

## Introduction

MPE is a rare benign variant of ependymoma that occurs most commonly in the cauda equina and filum terminale of the spinal cord (1,2). The majority of patients with MPEs are young adults and only a limited number of MPEs have been reported in elderly patients. MPEs are considered to represent grade I tumors characterized by slow growth. The tumor is often intradural, although local invasion and distant metastases are occasionally observed (3,4). The typical histopathological features of MPEs have been well described, whereas reports of hyaline changes and low cellular variants are rarely published. The current study presents an unusual case of MPE with marked hyaline degeneration in an elderly male. The clinicopathological observations of the case are described and analyzed. Written informed consent was obtained from the patient.

## Case report

### Clinical presentation and diagnosis

**Figure 1.**
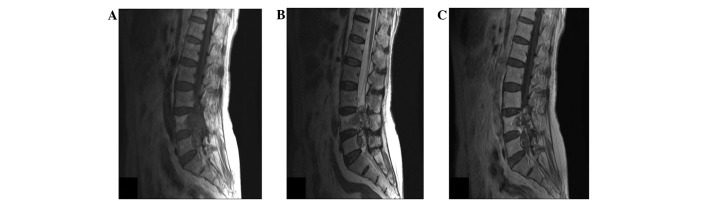
(A) T1-weighted sagittal image showing the hypointense lesion involving the L4 and L5 vertebrae with local bone destruction. (B) T2-weighted sagittal image showing the hyperintense lesion. (C) Post-contrast T1-weighted sagittal image showing the heterogeneous enhancement of the lesion.

A 72-year-old male presented with a 10-month history of lower back pain and dysesthesia at the lower abdominal level. The pain was of sudden onset with radiation to the legs. Weakness in the legs was also reported and the clinical signs were worsened by sitting. There was no history of trauma. The patient showed intermittent claudication after walking 200 m. Magnetic resonance imaging revealed a mass involving the L4 and L5 vertebrae with local bone destruction. The mass was hypointense on T1-weighted images, hyperintense on T2-weighted images and enhanced heterogeneously on post-contrast T1-weighted images (Fig. 1A–C). A total resection was performed and the patient’s post-operative course was uneventful. No subsequent adjuvant therapy was deemed necessary.

### Pathological analysis

**Figure 2.**
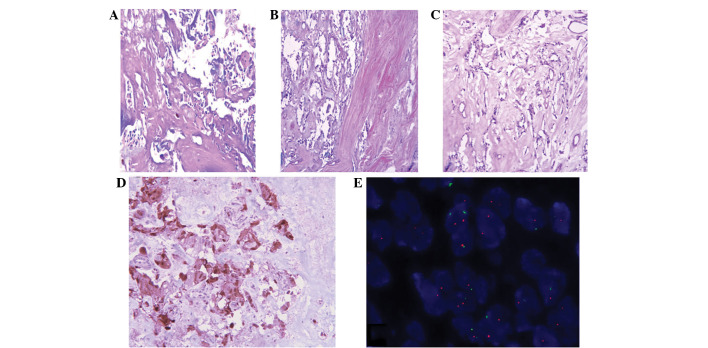
Photomicrographs of the tumor mass. (A) Acellular tumor mass with isolated neoplastic cells and a marked hyaline change observed in the blood vascular walls and stroma (hematoxylin and eosin; magnification, ×100). (B) Cystic and pseudopapillary component (hematoxylin and eosin; magnification, ×100). (C) Irregualr reticular neoplastic cells embedded in hyaline stroma (hematoxylin and eosin; magnification, ×100). (D) GFAP-positive neoplastic cells (immunoperoxidase; magnification, ×200). (E) Fluorescence *in situ* hybridization displaying two centromere 7 (green) and two EGFR (orange) signals. No amplification of the EGFR gene was observed. GFAP, glial fibrillary acidic protein; EGFR, epidermal growth factor receptor.

Microscopically, the tumor largely consisted of areas with low cellularity. In less cellular areas, marked hyaline changes were observed in the blood vascular walls and stroma (Fig. 2A). In other areas, the tumor showed pseudopapillary or reticular patterning formed by cuboidal cells on a hyaline background (Fig. 2B and C). Mitotic figures and necrosis were absent. The tumor cells showed marked positivity for glial fibrillary acidic protein (GFAP; Fig. 2D) and S-100 proteins, whereas the cells were negative for epithelial membrane antigen (EMA), cytokeratins (CK) and epidermal growth factor receptor (EGFR). The Ki-67 labeling index was ∼3%. Since EGFR may be a predictor of relapse in MPE (5), the EGFR gene was analyzed by fluorescence *in situ* hybridization. No amplification of the EGFR gene was observed (Fig. 2E). The results of pathological and immunohistochemical studies were consistent with the ependymal nature of neoplastic cells.

## Discussion

MPE is a benign variant of ependymoma that has a peak incidence between the third and fifth decades of life. It generally occurs in the filum terminale, but has also been identified in extra-spinal locations, including subcutaneous tissue and the brain (6,7). Clinical symptoms are directly associated with the mass location of the tumor. Histologically, it is typically characterized by papillae formed by the arrangement of cuboidal to columnar cells surrounding a central core, which contains blood vessels and myxoid change. The genesis of the stromal myxoid changes are indicative of an inundation of the plasma proteins generally located within blood vessels (8). In the current case, the pronounced deposition of hyaline materials was distributed among the tumor cells and within the blood vessel walls. Lim *et al* (9) previously hypothesized that the ‘proteinaceous’ deposits and hyaline vascular change were a result of increased vascular permeability that led to inundation of plasma proteins surrounding extravascular spaces. MPEs are generally slow-growing, therefore, the chronic long-standing anoxic conditions may lead to various regressive changes and necrosis. An additional explanation for the stromal change is the complicated structure of the conus medullaris and filum terminale, as this region is composed of an admixture of connective tissue, nerve fibers and neuroglia. It may be possible that these unique structural features result from the anatomical relationships of the MPE (10). Certain studies have indicated that, in specific circumstances, MPE cells produce basal lamina material, particularly in regions where ependymal cells are opposed to connective tissue (11,12). In the present case, tumor cells showed marked cytoplasmic staining for GFAP and S-100 protein, whereas immunoreactivity for CK, EMA, chromogranin, EGFR and synaptophysin were all negative. In addition, marked hyaline deposits were identified around the tumor cells, and pseudopapillary patterns or ependymal rosettes were rare. Therefore, in cases such as these, it is important to rule out the possibility of schwannomas, chordomas and metastatic mucinous adenocarcinomas. Spinal schwannomas appear on the myelin sheath of the spine and represent between one-quarter to one-third of all spinal tumors. Diagnostic features include a fibrous capsule and Antoni A and Antoni B areas. Similar to in MPEs, hyaline vessels and stroma are common in schwannomas and S100 is markedly expressed. However, GFAP is negative. It is extremely important to differentiate between a schwannoma and MPE prior to surgery, as an MPE has the potential to disseminate through the cerebrospinal fluid throughout the neuraxis and must be removed completely. The most common differential diagnosis at this location is that of a chordoma. The tumor cells are large with characteristic physaliferous cytoplasm, and the immunocytochemistry of CK is positive (13). Metastatic mucinous adenocarcinomas show epithelial cords and groups with an acinar arrangement. The cancer cells are more pleomorphic and are CK- and EMA-positive. An analysis using electron microscopy is extremely important for forming a differential diagnosis. Specific ultrastructural features, including microvilli, cilia, desmosomal attachments and cytoplasmic filaments, are indicative of a diagnosis of MPE (14,15).

In conclusion, specific regressive changes are observed in MPE and marked hyaline degeneration may lead to an acellular growth pattern, therefore, it is important to rule out the possibility of other tumors. The best curative treatment lies in a complete surgical resection, and long-term follow-up of the whole neuraxis must be performed.
